# Developing and evaluating a novel rapid test for recent HIV-1 infection: comparison with commercial LAg-EIA assays

**DOI:** 10.1128/jcm.01208-25

**Published:** 2025-12-29

**Authors:** Jing Liu, Ping Liu, Runhua Ye, Shitang Yao, Qiyu Zhu, Yan Jiang, Cong Jin

**Affiliations:** 1 National Key Laboratory of Intelligent Tracking and Forecasting for Infectious Diseases, National Center for AIDS/STD Control and Prevention, Chinese Center for Disease Control and Prevention, Beijing, China; 2 Department of AIDS Control and Prevention, Dehong Prefecture Center for Disease Control and Prevention, Mangshi, Yunnan, China; Mayo Clinic Minnesota, Rochester, Minnesota, USA

**Keywords:** HIV-1 recency infection, rapid test, LAg-EIA, MDRI, FRR

## Abstract

Accurate identification of recent HIV-1 infections is critical for real-time epidemic monitoring. However, conventional Limiting Antigen Avidity Enzyme Immunoassays (LAg-EIAs) are restricted to laboratory settings. A novel rapid recency test based on the limiting antigen avidity principle was developed for point-of-care use. We evaluated the performance of the rapid HIV-1 recency test using 500 longitudinal plasma specimens from 107 seroconverters. The mean duration of recent infection (MDRI) was estimated via binomial regression with maximum likelihood modeling. The false recent rate (FRR) was assessed using samples from individuals with long-term infection, including those on antiretroviral therapy (ART). Concordance was compared with two commercial LAg-EIA kits (Maxim and KingHawk). The rapid assay yielded an MDRI of 123 days (95% CI: 87–138), shorter than Maxim (152 days, 95% CI: 137–172) and KingHawk (131 days, 95% CI: 107–140). Among ART-naïve individuals infected for over 1 year, FRR was 5.3%, similar to Maxim (5.9%) and slightly higher than KingHawk (2.7%). High concordance was observed with Maxim (93.1%, kappa = 0.743) and KingHawk (89.4%, kappa = 0.558). In ART-treated individuals, FRR was significantly higher in the early ART group (69.9%) compared to the late ART group (20.2%, *P* < 0.001). The novel rapid HIV-1 recency assay demonstrates acceptable MDRI and FRR and strong agreement with commercial LAg-EIA kits. Its simplicity and rapid turnaround make it a promising tool for decentralized surveillance and targeted HIV interventions, especially in resource-limited settings.

**IMPORTANCE:**

Rapid detection of recent HIV-1 infections is essential for monitoring ongoing transmission and guiding targeted prevention efforts. However, currently used laboratory-based recency assays require specialized facilities and trained personnel, limiting their use in decentralized or resource-limited settings. In this study, we evaluated a newly developed rapid test that identifies recent HIV-1 infections within minutes using a simple, instrument-free format. The test showed strong agreement with two widely used laboratory assays and demonstrated performance suitable for surveillance applications. Its ease of use, rapid turnaround, and minimal infrastructure requirements make this rapid test a practical tool for expanding real-time HIV monitoring and improving the efficiency of public health responses.

## INTRODUCTION

Acquired immunodeficiency syndrome (AIDS) has remained a major global health challenge for over four decades, with the global burden of human immunodeficiency virus (HIV) infection continuing to grow. According to the latest report from the Joint United Nations Programme on HIV/AIDS (UNAIDS) (1), approximately 40.8 million individuals were living with HIV worldwide by the end of 2024, and 1.3 million new infections occurred that year. In response to the global goal of achieving the 95-95-95 targets, many countries have adopted innovative intervention strategies aimed at strengthening HIV prevention and control efforts (2–5).

Although routine monitoring of HIV prevalence provides valuable information on overall epidemic burden, such prevalence data cannot capture the real-time dynamics of new infections in subpopulations. Accurate estimation of HIV incidence is essential for tracking epidemic trends, assessing the effectiveness of prevention strategies, and identifying high-risk populations and transmission hotspots (6–9). However, determining the duration of infection based on routine surveillance data remains challenging, limiting the reliability of incidence estimates (10, 11). Although prospective cohort studies are considered the gold standard, they are costly, time-consuming, and prone to selection bias (12–14). Alternatively, mathematical modeling using retrospective data has been applied, but such approaches are also subject to inherent estimation bias (15).

Laboratory-based assays for identifying recent infections have become a widely accepted alternative for incidence estimation. These assays detect dynamic changes in one or more biomarkers during the early phase of HIV infection, allowing for differentiation between recent and long-term infections. For any HIV incidence assay, performance is generally characterized by both the mean duration of recent infection (MDRI) and the false recent rate (FRR) (16). Several assay formats have been developed (17–26), among which the BED-capture enzyme immunoassay (BED-CEIA) and the limiting antigen avidity enzyme immunoassay (LAg-EIA) have been commercialized and are widely utilized in surveillance programs. The LAg-EIA typically employs the recombinant immunodominant region of gp41 (rIDR-M) as the coating antigen (20), which provides cross-subtype reactivity and is sensitive to antibody maturation dynamics, making it the most commonly used antigen for avidity-based recency testing. By utilizing a limited amount of antigen, the assay restricts the binding of low-avidity antibodies and preferentially captures the high-avidity antibodies characteristic of long-term infections (27).

Since its introduction and subsequent localization in China in 2012, the LAg-EIA assay has been systematically evaluated for key performance parameters (28). It has since been incorporated into routine sentinel surveillance, molecular epidemiology investigations, and the screening of high-risk populations (29–31). However, the application of laboratory-based assays such as LAg-EIA requires trained personnel, specialized laboratory equipment, and cold-chain transportation, which limit their utility in point-of-care testing (POCT) settings and remote or resource-constrained regions.

**Fig 1:**
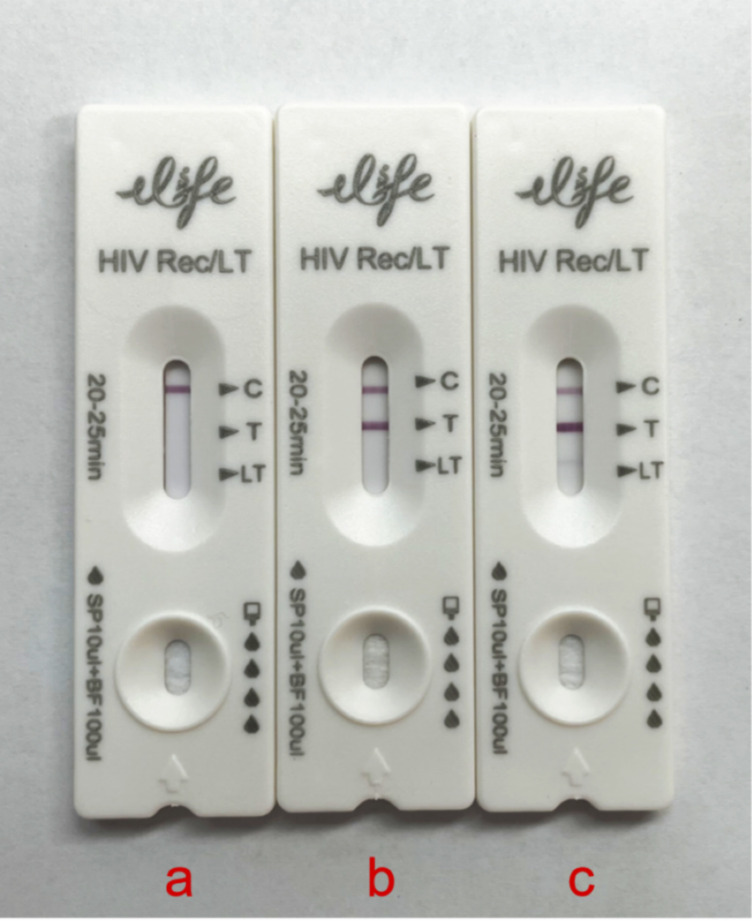
Interpretation of the rapid HIV-1 recency assay results is based on the presence or absence of specific lines. (**a**) C line only: HIV-1 negative; (**b**) C + T lines: recent HIV-1 infection; (**c**) C + T + LT lines: long-term HIV-1 infection.

To address the limitations of laboratory-based assays, a rapid HIV-1 recency test based on the LAg principle has been developed. This assay incorporates the core reaction components of LAg-EIA into an immunochromatographic platform, enabling a simplified, instrument-free workflow suitable for decentralized settings. The test strip contains three visually interpretable lines: a control line (C), a test line (T), and a long-term infection line (LT) (Fig. 1). Results are available within 20 mins, allowing for visual classification of HIV-1 negative and recent/long-term HIV-1 infections. The assay is designed to be incorporated into standard HIV diagnostic algorithms, supporting real-time epidemic monitoring and targeted intervention strategies.

In this study, we systematically evaluated the performance of the newly developed rapid test using plasma samples with well-defined duration of HIV-1 infection. Its performance was compared with two commercially available LAg-EIA kits widely used in China, focusing on two key parameters: MDRI and FRR. Additionally, we assessed the concordance across assays in classifying recent infections and investigated their FRR performance in individuals receiving antiretroviral therapy (ART).

## MATERIALS AND METHODS

### Samples collection

This study was conducted within a prospective cohort of HIV-negative individuals identified as being at high-risk for HIV infection between 2009 and 2018. Participants were followed at 3-month intervals, during which specimens were collected and diagnostic test results were recorded. HIV infection status was confirmed by Western blot (WB) using the HIV Blot 2.2 kit (MP Diagnostics, Singapore). A total of 500 longitudinal specimens from 107 individuals with estimated HIV-1 seroconversion dates were included for assay evaluation.

The date of seroconversion was estimated using the following criteria: (i) for individuals with only a last WB-negative and first WB-positive antibody test dates, the seroconversion date was defined as the midpoint between the two dates (32); (ii) for individuals with an indeterminate WB result followed by a WB-positive test, the midpoint was used if the interval was less than 14 days; otherwise, the seroconversion date was assigned as 7 days after the WB-indeterminate result (33); (iii) for individuals with last WB-negative, WB-indeterminate, and first WB-positive dates, the second approach described above was applied.

Based on study objectives, specimens were categorized into the following groups:

1. MDRI and FRR estimation group: A total of 303 specimens from 87 individuals with known durations post seroconversion, ranging from 6 to 3,443 days, were used to estimate the MDRI and the FRR.
2. ART-treated chronic infection group: A total of 197 plasma specimens were collected from 67 individuals infected with HIV-1 for more than 1 year and who had received ART. This group was further stratified into early ART initiation group (initiated ART within 1 year after seroconversion, *n* = 93) and late ART initiation group (initiated ART more than 1 year after seroconversion, *n* = 104).

### Rapid HIV-1 recency test assay

The rapid HIV-1 recency test evaluated in this study was co-developed by our laboratory and Sichuan Alife Biotechnology Co., Ltd. (Chengdu, China) and has since been manufactured and commercialized by Alife as a ready-to-use diagnostic kit.

#### Principle of the rapid HIV-1 recency assay

**Fig 2:**
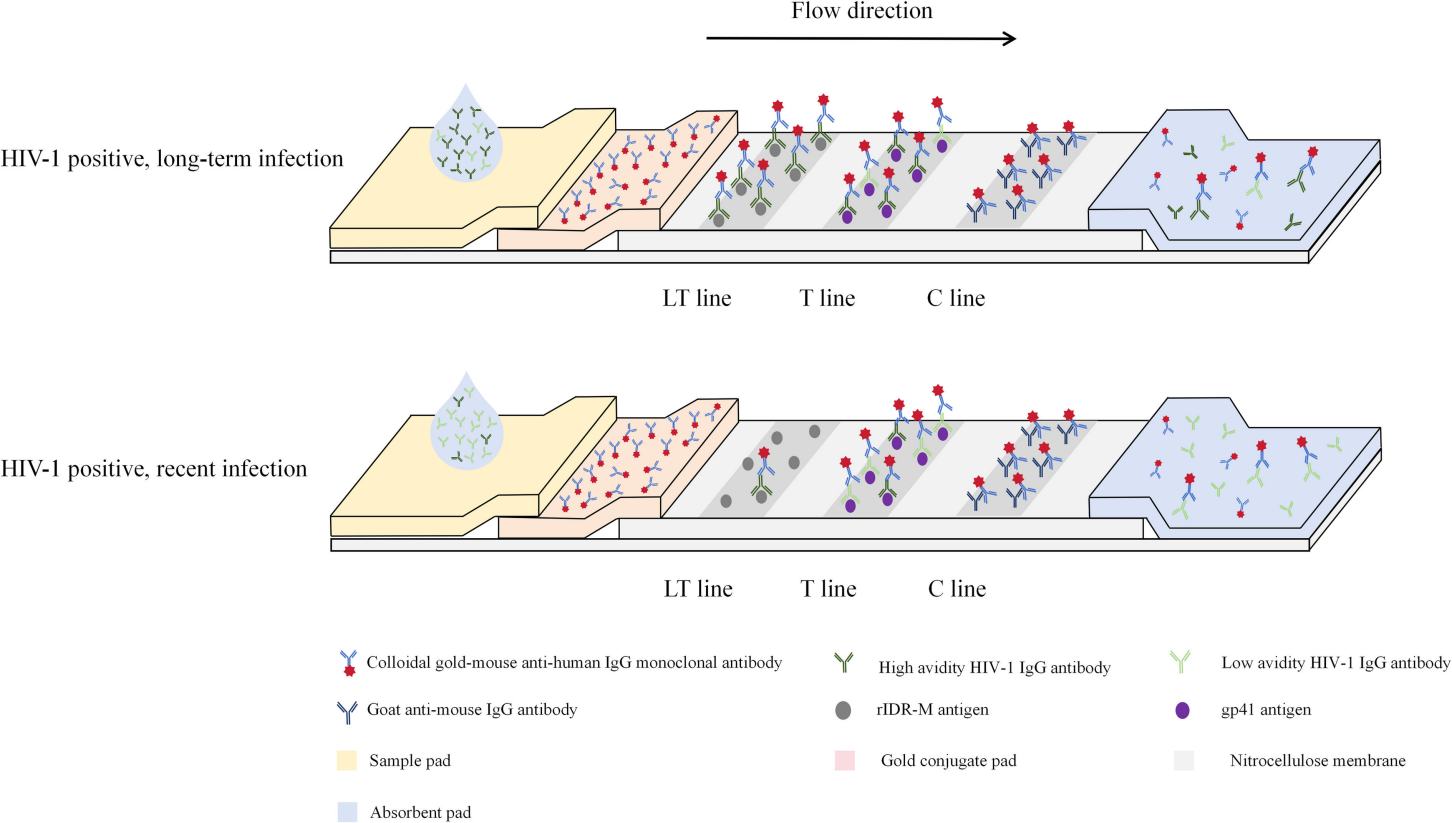
Schematic representation of the HIV-1 rapid recency assay based on colloidal gold immunochromatography. Samples migrate through the test strip by capillary action. The LT line contains recombinant rIDR-M antigen, the T line contains recombinant gp41 antigen, and the C line contains goat anti-mouse IgG antibody. High-avidity antibodies in long-term infections bind strongly to both gp41 and rIDR-M, producing three visible lines (LT, T, and C). In recent infections, only the T and C lines appear.

The rapid HIV-1 recency assay is a lateral flow immunochromatographic test designed to distinguish recent from long-term infections (Fig. 2). During testing, antibodies from the specimen bind to gold-conjugated mouse anti-human IgG and migrate along the strip. Recombinant gp41 immobilized on the T line captures HIV-1 specific antibodies, while rIDR-M on the LT line, pre-treated with an acidic buffer (pH 3.0), allows indirect assessment of antibody avidity. Under these conditions, low-avidity antibodies dissociate and fail to bind the LT line, whereas high-avidity antibodies remain bound and generate a visible signal. The C line coated with anti-mouse IgG serves as a control to confirm proper assay performance.

#### Preparation of assay components

The rIDR-M antigen was expressed and purified from an *E. coli* strain carrying the rIDR-M plasmid, which was authorized and supplied by the U.S. Centers for Disease Control and Prevention. The recombinant gp41 protein and goat anti-mouse IgG antibody were purchased from Fapon Biotech Inc. (Guangdong, China), and the mouse anti-human IgG monoclonal antibody was obtained from Cholun Medical Co., Ltd. (Shenzhen, China).

The assay components were systematically optimized to ensure stable performance and clear discrimination between recent and long-term HIV-1 infections. Specifically, the coating concentrations of rIDR-M and gp41 antigens were determined using well-characterized plasma specimens from individuals with confirmed recent and long-term HIV-1 infections, as well as HIV-1 negative samples (data not shown). In addition, the buffer composition, material selection, and sample dilution ratio were further refined to enhance signal consistency and assay robustness.

Mouse anti-human IgG monoclonal antibodies (12 μg) were conjugated to colloidal gold nanoparticles (1,000 µL) by stirring at room temperature for 5 min. To block unoccupied protein-binding sites, 100 µL of 1% bovine serum albumin (BSA) solution was added and incubated for an additional 5 min. Following centrifugation, the pellet was resuspended in 0.1 M Tris buffer (pH 9.0) containing 6.7% sucrose and 0.3% casein, and the mixture was evenly applied onto the gold conjugate pads, which contained no additional reagents. The pads were then dried at 45°C under 10%–30% relative humidity.

The rIDR-M protein was diluted to 0.28 mg/mL in 10 mM sodium citrate buffer (pH 3.0), the recombinant gp41 protein was diluted to 1 mg/mL in 0.1 M Tris buffer (pH 8.0), and goat anti-mouse IgG antibodies were diluted to 1 mg/mL in 0.1 M phosphate buffer (pH 8.0). These reagents were dispensed onto nitrocellulose membranes using an automated membrane dispenser to form the LT, T, and C lines. The membranes were then dried at 32°C for 18–22 h under controlled humidity (10%–30%). The sample pad was pre-treated with 0.1 M phosphate buffer (pH 7.2) containing 20% fetal bovine serum and 0.25% Tween-80 and then dried and stored under low-humidity conditions (<30%). All test components, including sample pad, conjugate pad, nitrocellulose membrane, and absorbent pad, were assembled onto an adhesive backing card in accordance with standard operating procedures and stored at 4–30°C with a relative humidity below 25% until use. Under these storage conditions, the validity period of the test strip is 24 months.

### Laboratory procedures

The rapid HIV-1 recency test was conducted independently by two trained laboratory technicians blinded to the sample background information. When discrepant interpretations occurred, the strip was reviewed by a third senior investigator, and the final result was based on two concordant interpretations. A volume of 5 μL of plasma was mixed with 50 μL of assay dilution buffer and applied to the test strip. After incubation at room temperature for 20 min, results were visually interpreted according to the following criteria (Fig. 1): (a) C line Only: HIV-1 negative; (b) C + T lines: recent HIV-1 infection; (c) C + T + LT lines: long-term HIV-1 infection.

For comparison, two commercial HIV-1 LAg-EIA kits, the Maxim HIV-1 LAg-Avidity EIA (Maxim Biomedical, Inc., Rockville, MD, USA) and the Kinghawk HIV-1 LAg-Avidity EIA (Beijing Kinghawk Pharmaceutical Co., Ltd., Beijing, China), were performed strictly following the manufacturers’ protocols. Briefly, both assays use a solid-phase format with a limited concentration of HIV-1 recombinant antigen (rIDR-M) coated onto microplate wells. Diluted plasma specimens were added to the wells and incubated for 60 min, followed by a 15-min treatment with dissociation buffer to remove low-avidity antibodies. Horseradish peroxidase (HRP)-conjugated goat anti-human IgG was then added and incubated for 30 min. Subsequently, TMB substrate was added and incubated for 15 min before stopping the reaction with stop solution. Optical density (OD) was measured at 450 nm, and the results were normalized using the internal calibrators provided in each kit to obtain the normalized optical density (ODn). According to the manufacturers’ instructions, an ODn ≤ 1.5 was classified as recent infection, while an ODn > 1.5 as long-term infection for both Maxim and KingHawk assays.

To minimize the impact of freeze-thaw cycles, plasma samples were pre-aliquoted during initial processing, and each tube was thawed no more than three times. Separate aliquots were used for the rapid recency test and for the two LAg-EIA assays.

#### Estimation of MDRI and FRR

MDRI is defined as the average time an individual remains classified as recently infected after HIV seroconversion. Several statistical approaches have been developed for MDRI estimation (28, 34). In this study, MDRI was estimated using binomial regression with a maximum likelihood approach, an approach validated in multiple epidemiological contexts (32). Two functional forms (cloglog_linear and logit_cubic) were applied to characterize the full early antibody-maturation curve, and models were fitted to specimens collected within 800 days post-seroconversion. To ensure reliable curve fitting, specimens were excluded if the interval between the last HIV WB-negative and first WB-positive result exceeded 365 days (32), leaving 150 valid samples for analysis. Using the formula:


$$\text{MDRI=}\int_{\text{0}}^{\text{t}}{\text{P}}^{\text{R}}\text{(}t)\;dt$$

Following WHO/UNAIDS guidance, a cut-off of *T* = 2 years was used for MDRI estimation (16, 34). All analyses were performed with the R package *inctools* (https://cran.r-project.org/web/packages/inctools/).

FRR was defined as the proportion of specimens from individuals infected for more than *T* days that were misclassified as recent. In this study, FRR was evaluated for samples with time post-seroconversion >1 year, >2 years, and >2 × MDRI.

### Statistical analysis

Estimation of MDRI was performed using R(version 4.4.1). FRR analyses and other statistical comparisons were conducted using SPSS (version 29.0; IBM Corp., Armonk, NY, USA). Figures were generated using GraphPad Prism (version 10; GraphPad Software, San Diego, CA, USA). A two-tailed *P*-value of <0.05 was considered statistically significant.

## RESULTS

### Antibody avidity dynamics measured by LAg-EIA kits

**Fig 3:**
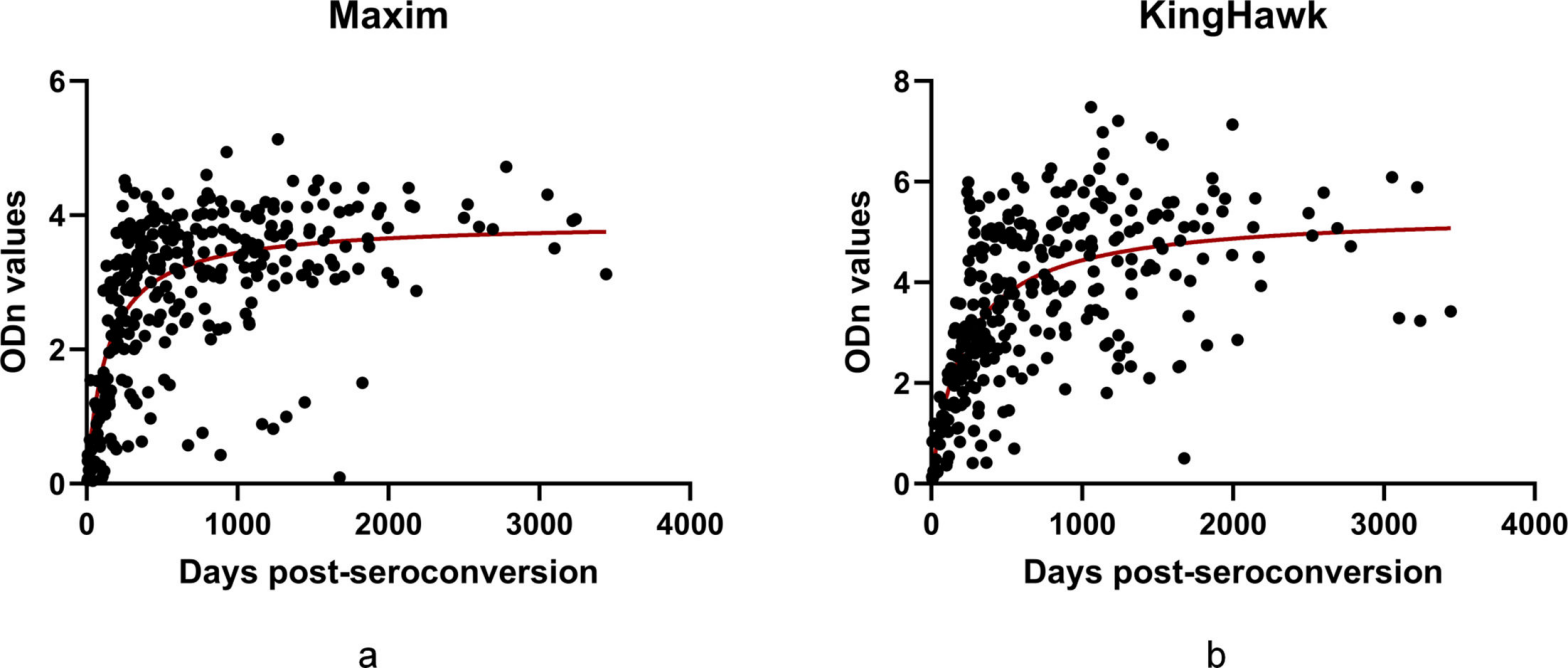
Scatter plots showing the relationship between ODn values and days post-seroconversion for two LAg-EIA assays: (**a**) Maxim and (**b**) KingHawk. Each point represents a sample, with ODn values plotted on the *y*-axis and days post-seroconversion on the *x*-axis. A smooth red line indicates the trend of the data. The plots demonstrate the increasing ODn values over time since seroconversion.

The kinetics of antibody avidity among 87 HIV-1 seroconverters, as measured by the Maxim and KingHawk LAg-EIA kits, are shown in Fig. 3. Antibody avidity increased rapidly during the first 200 days following seroconversion and then gradually decelerated and reached a plateau. Beyond 1,000 days post-seroconversion, avidity values remained stable, with only a few outlier samples exhibiting unusually low readings. Compared to the KingHawk assay, the Maxim assay demonstrated a slightly slower rate of avidity increase over time.

### MDRI estimates for rapid HIV-1 recency test and LAg-EIA assays

**Fig 4:**
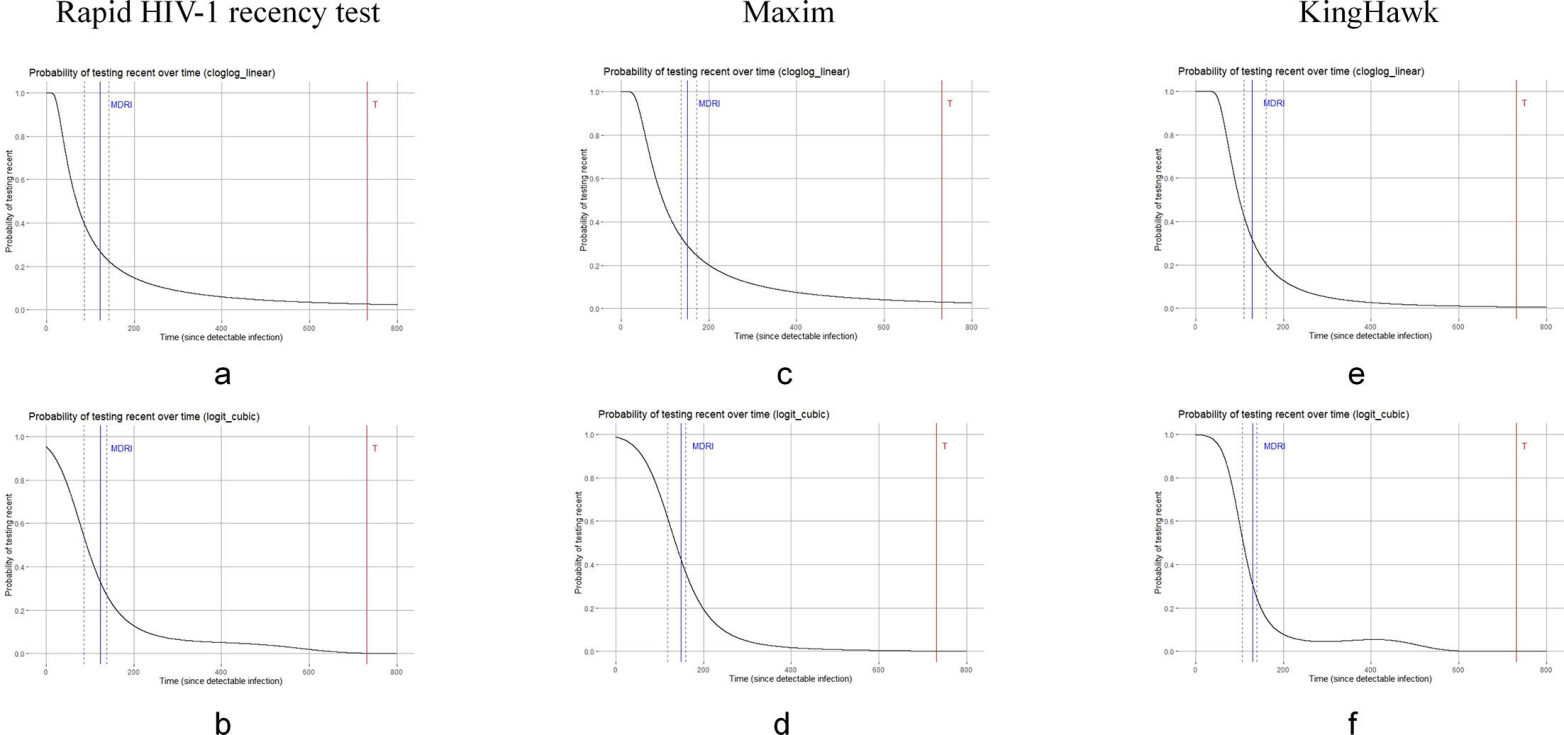
MDRI estimation curves for the rapid HIV-1 recency assay and LAg-EIA assays, based on two different models. Panels (**a**) and (**b**) show the MDRI curves for the rapid HIV-1 recency assay using the cloglog_linear model and logit_cubic model, respectively. Panels (**c**) and (**d**) represent the Maxim assay with the same models. Panels (**e**) and (**f**) represent the KingHawk assay. In all panels, the blue vertical dashed lines represent the MDRI, and the red vertical lines represent the time cut-off used for MDRI estimation (*T* = 2 years).

Following data filtering in R, a total of 150 plasma samples collected within 800 days post-seroconversion were included in the MDRI estimation (Fig. 4). For the rapid HIV-1 recency test, the MDRI was estimated at 122 days (95% CI: 86–142) using the cloglog_linear model and 123 days (95% CI: 87–138) with the logit_cubic model. For the Maxim assay, MDRI estimates were 152 days (95% CI: 137–172) and 149 days (95% CI: 118–159) using the cloglog_linear and logit_cubic models, respectively. The corresponding MDRI estimates for the KingHawk assay were 130 days (95% CI: 111–161) and 131 days (95% CI: 107–140). Final MDRI values for each assay were selected based on the model yielding the narrowest 95% confidence interval (Table 1).

**TABLE 1:** Estimated MDRI for the rapid recency test and two LAg-EIA assays (*n* = 150)

Assays	cloglog_linear		logit_cubic	
	MDRI (days, SD)	95% CI (days)	MDRI (days, SD)	95% CI (days)
Rapid HIV-1 recency test	122 (17.6)	(86, 142)	123 (17.0)	(87, 138)
Maxim	152 (11.9)	(137, 172)	149 (14.2)	(118, 159)
KingHawk	130 (15.0)	(111, 161)	131 (12.6)	(107, 140)

### FRR estimates for rapid HIV-1 recency test and LAg-EIA assays

The FRR was evaluated using specimens from long-term infected individuals. Among 124 samples collected more than 2 years post-seroconversion, five were misclassified as recent by the rapid assay, resulting in an FRR of 4.0% (5/124) (Table 2). In 187 samples collected more than 1 year after seroconversion, 10 were misclassified, yielding an FRR of 5.3% (10/187). When using a threshold of more than twice the estimated MDRI (234 samples), the FRR slightly increased to 6.0% (14/234).

**TABLE 2:** Estimated FRR for the rapid HIV-1 recency test and two LAg-EIA assays under different time cut-offs

Assays	Time cut-off	FRR (% [*n*/*N*])
Rapid HIV-1 recency test	>2 years	4.0% (5/124)
	>1 year	5.3% (10/187)
	>2 × MDRI (MDRI = 123 days)	6.0% (14/234)
Maxim	>2 years	5.6% (7/124)
	>1 year	5.9% (11/187)
	>2 × MDRI (MDRI = 152 days)	6.6% (14/212)
	>2 × MDRI (manual recommendation, MDRI = 161 days)	6.3% (13/205)
KingHawk	>2 years	0.8% (1/124)
	>1 year	2.7% (5/187)
	>2 × MDRI (MDRI = 131 days)	4.4% (10/227)
	>2 × MDRI (manual recommendation, MDRI = 130 days)	4.4% (10/227)

For comparison, the commercial LAg-EIA assays showed similar patterns. The Maxim assay produced FRRs of 5.6% (7/124) for specimens more than 2 years post-seroconversion, 5.9% (11/187) for more than 1 year, and 6.6% (14/212) when applying a threshold of more than 2 × MDRI (MDRI = 152 days) (Table 2). The KingHawk assay showed lower rates, with FRRs of 0.8% (1/124) for more than 2 years, 2.7% (5/187) for more than 1 year, and 4.4% (10/227) when applying a threshold of more than 2 × MDRI (MDRI = 131 days) (Table 2).

### Concordance between the rapid HIV-1 recency test and LAg-EIA assays

Among 303 ART-naïve specimens, the rapid HIV-1 recency assay classified 43 as recent infections, while the Maxim and KingHawk assays identified 54 and 41 recent cases, respectively. The concordance between the rapid assay and the Maxim assay was 93.1% (Kappa = 0.743, *P* < 0.05) (Table 3) and 89.4% (Kappa = 0.558, *P* < 0.05) when compared with the KingHawk assay (Table 4). The agreement between the Maxim and KingHawk assays was 90.4% (Kappa = 0.639, *P* < 0.05) (Table 5).

**TABLE 3:** Comparing rapid HIV-1 recency test with Maxim assay for classification of recent or long-term infections

Rapid HIV-1 recency test	Maxim		Total
	Long-term	Recent	
Long-term	244	16	260
Recent	5	38	43
Total	249	54	303
Concordance = 93.1%; Kappa = 0.743 (*P* < 0.05)			

**TABLE 4:** Comparing rapid HIV-1 recency test with KingHawk assay for classification of recent or long-term infections

Rapid HIV-1 recency test	KingHawk		Total
	Long-term	Recent	
Long-term	245	15	260
Recent	17	26	43
Total	262	41	303
Concordance = 89.4%; Kappa = 0.558 (*P* < 0.05)			

**TABLE 5:** Comparing Maxim assay with KingHawk assay for classification of recent or long-term infections

KingHawk	Maxim		Total
	Long-term	Recent	
Long-term	241	21	262
Recent	8	33	41
Total	249	54	303
Concordance = 90.4%; Kappa = 0.639 (*P* < 0.05)			

### FRR among ART-treated individuals with long-term infection

Among the 93 specimens collected from individuals with long-term HIV-1 infection who initiated ART within one year of seroconversion (early ART group), the rapid HIV-1 recency assay misclassified 65 specimens as recent, corresponding to an FRR of 69.9% (65/93) (Table 6). In comparison, among the 104 specimens from individuals who initiated ART more than one year post-seroconversion (late ART group), 21 specimens were misclassified, yielding an FRR of 20.2% (21/104) (Table 6). The median time from seroconversion to ART initiation was 147 days (IQR, 59–224) for the early ART group and 1,236 days (IQR, 898–1,841) for the late ART group. The FRR in the late ART group was significantly lower than that in the early ART group (*P* < 0.001) (Table 6). Notably, within both groups, no statistically significant association was observed between FRR and the duration of ART. FRR estimates for the Maxim and KingHawk assays in the same cohort are shown in Table 6. Overall, early ART initiation was consistently associated with higher FRRs across all assays evaluated.

**TABLE 6:** Comparison of FRRs between early and late ART initiation groups

	Rapid test for HIV recent infection		Maxim		KingHawk	
	FRR (% [*n*/*N*])	*P* value	FRR (% [*n*/*N*])	*P* value	FRR (% [*n*/*N*])	*P* value
Early initiation ART	69.9% (65/93)	＜0.001^*a*^	64.5% (60/93)	＜0.001^*a*^	60.2% (56/93)	＜0.001^*a*^
Duration of ART < 1 year	60.0% (9/15)	0.545	53.3% (8/15)	0.323	53.3% (8/15)	0.552
Duration of ART ≥ 1 year	71.8% (56/78)		66.7% (52/78)		61.5% (48/78)	
Duration of ART < 2 year	68.6% (35/51)	0.769	66.7% (34/51)	0.633	58.8% (30/51)	0.763
Duration of ART ≥ 2 year	71.4% (30/42)		61.9% (26/42)		61.9% (26/42)	
Duration of ART < 3 year	67.2% (39/58)	0.473	65.5% (38/58)	0.795	58.6% (34/58)	0.686
Duration of ART ≥ 3 year	74.3% (26/35)		62.9% (22/35)		62.9% (22/35)	
Late initiation ART	20.2% (21/104)		9.6% (10/104)		9.6% (10/104)	
Duration of ART < 1 year	27.3% (3/11)	0.825	9.1% (1/11)	1	18.2% (2/11)	0.632
Duration of ART ≥ 1 year	19.4% (18/93)		9.7% (9/93)		8.6% (8/93)	
Duration of ART < 2 year	23.1% (9/39)	0.57	7.7% (3/39)	0.864	10.3% (4/39)	1
Duration of ART ≥ 2 year	18.5% (12/65)		10.8% (7/65)		9.2% (6/65)	
Duration of ART < 3 year	17.5% (11/63)	0.39	6.3% (4/63)	0.289	7.9% (5/63)	0.704
Duration of ART ≥ 3year	24.4% (10/41)		14.6% (6/41)		12.2% (5/41)	

^*a*^ Early initiation ART vs late initiation ART.

## DISCUSSION

In this study, we systematically evaluated a novel rapid assay for identifying recent HIV-1 infections based on the Limiting Antigen Avidity principle. The assay demonstrated robust performance in distinguishing recent from long-term infections and showed strong concordance with two widely used commercial LAg-EIA kits. These findings support its applicability for routine use in both surveillance systems and diagnostic workflows.

The MDRI and FRR are two key performance indicators for assays designed to detect recent HIV-1 infection. An optimal assay should achieve an adequate MDRI while maintaining a low FRR to ensure accurate incidence estimation (35). In our evaluation, the novel rapid assay yielded an MDRI of 123 days (95% CI: 87–138), which was shorter than that of the Maxim (152 days, 95% CI: 137–172) and KingHawk (131 days, 95% CI: 107–140) assays. These results are consistent with previous reports and manufacturer-provided values (28, 36). Regarding FRR, the rapid assay misclassified 5.3% of specimens collected more than 1 year post-seroconversion, a rate comparable to the Maxim assay (5.9%) and slightly higher than KingHawk (2.7%). Since elevated FRR can lead to overestimation of HIV incidence by incorrectly classifying chronic infections as recent, assay-specific validation remains essential. In view of the differences in key parameters and assay formats across recency testing methods, WHO recommends that surveillance systems maintain methodological consistency within the same population and time period (37). Such consistency improves the accuracy of incidence estimation and strengthens the value of surveillance data for public health decision-making.

LAg-EIA assays are widely used for HIV incidence estimation due to their high accuracy and reproducibility. However, their implementation is limited by several operational constraints, including the need for specialized laboratory infrastructure, cold-chain storage, prolonged assay time, and trained personnel. These factors hinder their application in POCT, especially in remote or resource-limited settings. In contrast, the immunochromatographic rapid assay developed in this study offers several practical advantages. It requires minimal equipment, delivers results within 20 min, and allows for visual interpretation without instrumentation. These characteristics make it particularly suitable for field-based surveillance and decentralized primary healthcare facilities.

We further assessed assay performance among individuals with long-term HIV-1 infection receiving ART. The rapid assay showed a substantially higher FRR in the early ART group (69.9%) compared to the late ART group (20.2%). This disparity likely reflects the impact of early ART initiation on the maturation of HIV-specific antibodies. Early viral suppression reduces antigen exposure and truncates somatic hypermutation, leading to incomplete avidity development and persistent misclassification as recent (38), whereas delayed ART allows more complete affinity maturation before treatment begins (39, 40). Interestingly, in late ART initiators, the rapid assay yielded higher FRRs than the LAg-EIA assays, which we attribute to the greater precision of the LAg-EIA platforms rather than to ART-induced changes in antibody avidity. Given the impact of ART on FRR, WHO recommends that ART-experienced individuals be excluded from incidence estimation based on recency testing (41). Future studies should investigate the potential impact of PrEP exposure and other conditions that alter or modulate immune responses on the performance and accuracy of rapid recency assays.

China has established a robust sentinel surveillance system for HIV monitoring, targeting key populations such as people who inject drugs, men who have sex with men (MSM), female sex workers, and STI clinic attendees (42, 43). Integrating the newly developed rapid recency assay into such systems could enhance detection efficiency, broaden population coverage, and facilitate timely responses to emerging transmission clusters. Despite its modest FRR, the assay’s simplicity, speed, and cost-effectiveness make it particularly suitable for decentralized or resource-limited settings. In contrast to the Determine HIV-1/2 Ag/Ab Combo assay, which detects HIV antibodies and p24 antigen to identify acute and chronic phases of infection but provides no information on infection duration after seroconversion (44, 45), rapid recency assay measures antibody avidity to classify infections as recent or long term. These fundamental differences underscore the complementary roles between conventional HIV screening assays and the newly developed rapid recency assays: screening assays serve as frontline diagnostic tools for identifying HIV infection, whereas rapid recency assays are designed to support surveillance systems and estimate population-level incidence.

This study has several limitations. First, the specimens were derived from a specific prospective cohort, which may limit the generalizability of the findings to other populations or epidemiological contexts. Second, we did not perform the HIV-1 subtype characterization. Therefore, the assay’s performance across diverse subtypes remains to be evaluated in future studies. Third, the assay is designed for use only in individuals confirmed as HIV-1 seropositive by WB. It should not be used in seronegative individuals or in those with acute infections (p24 antigen/RNA positive but antibodies negative).

In summary, the novel rapid HIV-1 recency assay demonstrated favorable performance in terms of MDRI, FRR, and concordance with existing LAg-EIA assays. Its operational simplicity, rapid turnaround, and field adaptability make it a promising tool for real-time HIV surveillance and targeted intervention. Further validation in diverse populations and integration of digital interpretation tools, such as artificial intelligence, may enhance its accuracy, reproducibility, and applicability in field-based programs. Moreover, investigating the potential clinical utility of recency testing, either as an adjunct to patient management or to guide individualized therapeutic strategies, could extend its value beyond surveillance.
